# Intrahemispheric Perfusion in Chronic Stroke-Induced Aphasia

**DOI:** 10.1155/2017/2361691

**Published:** 2017-03-05

**Authors:** Cynthia K. Thompson, Matthew Walenski, YuFen Chen, David Caplan, Swathi Kiran, Brenda Rapp, Kristin Grunewald, Mia Nunez, Richard Zinbarg, Todd B. Parrish

**Affiliations:** ^1^Center for the Neurobiology of Language Recovery, Northwestern University, Evanston, IL, USA; ^2^Department of Communication Sciences and Disorders, School of Communication, Northwestern University, Evanston, IL, USA; ^3^Department of Neurology, Feinberg School of Medicine, Northwestern University, Evanston, IL, USA; ^4^Department of Radiology, Feinberg School of Medicine, Northwestern University, Evanston, IL, USA; ^5^Massachusetts General Hospital, Department of Neurology, Harvard Medical School, Boston, MA, USA; ^6^Department of Speech, Language, and Hearing, College of Health & Rehabilitation, Boston University, Boston, MA, USA; ^7^Department of Cognitive Science, Krieger School of Arts & Sciences, Johns Hopkins University, Baltimore, MD, USA; ^8^Department of Psychology, Weinberg College of Arts and Sciences, Northwestern University, Evanston, IL, USA

## Abstract

Stroke-induced alterations in cerebral blood flow (perfusion) may contribute to functional language impairments and recovery in chronic aphasia. Using MRI, we examined perfusion in the right and left hemispheres of 35 aphasic and 16 healthy control participants. Across 76 regions (38 per hemisphere), no significant between-subjects differences were found in the left, whereas blood flow in the right was increased in the aphasic compared to the control participants. Region-of-interest (ROI) analyses showed a varied pattern of hypo- and hyperperfused regions across hemispheres in the aphasic participants; however, there were no significant correlations between perfusion values and language abilities in these regions. These patterns may reflect autoregulatory changes in blood flow following stroke and/or increases in general cognitive effort, rather than maladaptive language processing. We also examined blood flow in perilesional tissue, finding the greatest hypoperfusion close to the lesion (within 0–6 mm), with greater hypoperfusion in this region compared to more distal regions. In addition, hypoperfusion in this region was significantly correlated with language impairment. These findings underscore the need to consider cerebral perfusion as a factor contributing to language deficits in chronic aphasia as well as recovery of language function.

## 1. Introduction

Recovery of language in chronic stroke-induced aphasia involves recruitment of undamaged tissue in the contralesional (typically right) and/or the ipsilesional hemisphere of the brain [1–4]. Although it has been suggested that ipsilesional, and even perilesional, tissue is best suited to support recovery, there are several factors that influence recruitment of undamaged tissue during functional recovery, including poststroke alterations in vascular physiology.

Emerging evidence from multiple sources suggests that restoration of cerebral blood flow (the rate at which blood perfuses a neural region) is critically associated with functional recovery. Blood delivers oxygen and glucose to the brain that is required for aerobic metabolism supporting neural activity [5]. In hyperacute stages of stroke, cortical spreading depression originating from the infarction site causes the lesion to expand [6], which affects symptom severity [7]. In addition, perilesional tissue becomes inflamed [8]. A settling of these events (e.g., lesion stabilization, reduced inflammation) contributes to recovery of function in acute stroke-induced aphasia, when perfusion is most likely to reverse to prestroke levels, either spontaneously or through pharmacological interventions [9, 10].

Prestroke perfusion levels, however, may not be regained in all regions of the brain, leaving uninfarcted tissue hypoperfused well past the acute stage. Using arterial spin labeling MRI, Richardson et al. [11] found reduced perfusion values in the left (ipsilesional) hemisphere compared to the right hemisphere in 17 patients with chronic aphasia [11, 12] (see Table 1 for a review of studies of perfusion in chronic aphasia). Notably, negative correlations between perfusion and lesion volume were reported, with larger infarcts corresponding to greater interhemispheric differences in perfusion. However, the duration of aphasic symptoms (time since stroke) did not correlate with reduced perfusion, suggesting a stable state of chronic hypoperfusion in chronic aphasia [11].

Regions of hypoperfused but otherwise intact tissue can create what are essentially functional lesions in chronic stroke, where the neurons are viable but unable to sufficiently support processing [13–15]. Evidence from animal models indicates that although neurons survive with perfusion levels greater than about 10% of normal, neuronal function is compromised when perfusion levels are below roughly 30% of normal [13, 16]. In human adults, normal cerebral blood flow in gray matter ranges from 37 to 64 mL/100 g/min, and lower perfusion may preclude normal functioning [14]. Consideration of hypoperfused regions, therefore, offers an important refinement to the traditional lesion method used to make inferences about structure-function correspondences in the brain [17], in that impaired language functioning may result not only from regions directly lesioned by stroke, but also from “hibernating” hypoperfused regions [18–20]. For example, Love et al. [15] reported hypoperfusion of otherwise spared tissue in the left angular and supramarginal gyri associated with impaired reading in a chronic aphasic patient.

Hypoperfused neural tissue also may not be viable for support of language recovery; rather, regions with lesser reductions or uncompromised cerebral blood flow (rCBF) may be better candidates for treatment-induced upregulation of neural activity. For example, Thompson et al. [19] found that baseline perfusion was higher (i.e., nearer normal levels) in regions that showed upregulation of neural activity in patients who underwent treatment for agrammatism. Fridriksson et al. [20] found a similar pattern in 30 patients who received treatment for anomia: pretreatment perfusion levels in undamaged regions within the left hemisphere language network (excluding infarcted and perilesional regions) predicted patients' naming accuracy, suggesting that higher baseline cerebral blood flow may be related to the potential for a better treatment outcome.

Reduction in cerebral blood flow also alters hemodynamic autoregulation aimed at maximizing the delivery of oxygen by increasing the blood volume or oxygen extraction fraction [21]. This has a fundamental effect on the shape and timing of the hemodynamic response used to measure blood oxygenation level-dependent (BOLD) task changes [12]. If not taken into account, an abnormal hemodynamic response may lead to underestimation and/or inaccurate measurement of the BOLD signal in functional magnetic resonance imaging (fMRI) [19, 22, 23]. Bonakdarpour et al. [24] found that three of five individuals with chronic stroke aphasia showed a delayed hemodynamic response (delayed blood flow) in left perisylvian, relative to the left occipital, cortex during a lexical decision task. No such delay was seen in right perisylvian regions. Likewise, increased time-to-peak was seen in the five patients in left perilesional tissue during an overt naming task, but not in homologous right hemisphere regions. These delays correlated positively with lesion size (longer delays were seen in individuals with larger lesions) and negatively with aphasia severity as estimated using the Western Aphasia Battery-Revised (WAB-R) [25] (longer delays in individuals with more severe aphasia).

One region suggested to be particularly important for recovery of function is perilesional tissue. In rodent models, perilesional tissue undergoes neurophysiological changes, such as vascular proliferation and remodeling (angiogenesis) [26–29], reduced dendritic complexity, spine density, and synapses [26, 28, 30], and elevated rates of axonal sprouting [31, 32]. Hypoperfusion and reduced glucose metabolism also are prevalent in perilesional space [31, 33]. Notably, reversal to more normal neurophysiology within this region has been shown to coincide with recovery of function in animals as well as in acute phases of aphasia recovery in humans [34–37]. Presently, however, few studies have examined perfusion and/or reperfusion in perilesional tissue in chronic aphasia. Furthermore, within the aphasia literature there has been little research focused on what constitutes perilesional tissue and/or its role in recovery of language. In one study, Richardson [22] found reduced perfusion levels in individuals with chronic aphasia in a perilesional region of interest (ROI), defined as tissue from 3 to 8 mm surrounding the lesion, compared to its right hemisphere homologue.

In sum, prior evidence underscores the importance of examining perfusion in individuals with aphasia into the chronic stage, to augment understanding of the neural basis of language processing following stroke, and to determine the relation between perfusion and recovery of function. This paper examined perfusion in a group of individuals with chronic aphasia induced by left hemisphere ischaemic stroke and a cohort of healthy control participants. We tested between-subjects (aphasic versus healthy participants) differences in perfusion values in the left versus right hemisphere as well as in 38 ROIs in each hemisphere of the brain. We also examined perfusion in the patient group in perilesional ROIs, compared both to right hemisphere homologous regions and to remaining gray matter tissue in the left hemisphere (i.e., unlesioned, outside of the perilesional region). Perfusion was also examined in relation to scores on behavioral language tests reflecting overall aphasia severity, single word production (i.e., naming) and comprehension, spelling, and sentence production and comprehension ability. Finally, we examined perfusion in relation to individual and stroke-specific factors, including sex, age, education, lesion age (i.e., time post stroke), and lesion size (volume).

Overall, we expected reduced perfusion values in left hemisphere (ipsilesional) regions, but not in right hemisphere tissue in participants with aphasia relative to healthy controls. Also, based on prior studies with this clinical population, we expected perilesional perfusion to be reduced compared to the remainder of the left hemisphere and to correlate with lesion volume and aphasia severity, but not with lesion age, sex, education, or age.

## 2. Method

### 2.1. Participants

We tested 35 participants with aphasia subsequent to a single left hemisphere ischaemic stroke and 16 healthy adult controls (see Table 2). Participants with aphasia, presenting with anomia, dysgraphia, and agrammatism, were recruited from three research sites, Northwestern University (*n* = 9), Boston University and Massachusetts General Hospital (*n* = 21), and Johns Hopkins University (*n* = 5), respectively, as part of a large-scale NIDCD funded Clinical Research Center. Healthy controls were recruited from the greater Chicago area and tested at Northwestern University. The study was approved by the Institutional Review Boards of all three universities and all participants provided informed consent.

All participants were right-handed native English speakers. Participants with aphasia were older (range = 41–79 years; M = 57.7 years) than the healthy controls (range = 24–57 years; M = 32.3 years; two-sample, unequal variance *t*-test: *t*(36) = 9.19, *p* < .0001) and had fewer years of education (M = 15.8 versus M = 17.7 years; two-sample unequal variance *t*-test: *t*(32) = 3.33, *p* = .001). Participants with aphasia passed vision and hearing screenings (pure-tone audiometric screening at 40 dB, 1000 Hz) and had no other diagnosed brain disorders and no history of drug or alcohol abuse. Healthy controls had self-reported normal or corrected-to-normal vision and hearing and no history of speech, language, or learning disorders or substance abuse.

Participants with aphasia were all in the chronic stage and were at least twelve months post-stroke-onset (M: 59.3 months, SD: 53.0, range: 12–209 months). The diagnosis and overall severity of aphasia were based on administration of the Western Aphasia Battery-Revised (WAB-R) [25]; WAB-AQ scores ranged from 11.7 to 95.2 (M: 66.2, SD: 22.7). We also characterized each participant's language abilities using a battery of language tests, which included measures of spoken and written comprehension and production of words and sentences. Single word production and comprehension were tested using 26 items from the Confrontation Naming (CN) and Auditory Comprehension (AC) subtests of the Northwestern Naming Battery (NNB) [38] (10 low frequency nouns from the “Other” category on the NNB and 16 verbs). We used the Psycholinguistic Assessments of Language Processing in Aphasia (PALPA) [39] to evaluate spelling-to-dictation of words with high and low frequency (subtest 40). Finally, the Sentence Production Priming Test (SPPT) and the Sentence Comprehension Test (SCT) from the Northwestern Assessment of Verbs and Sentences (NAVS) [40], which include 30 items each to test canonical and noncanonical structures, were used to evaluate production and comprehension of sentences of different syntactic complexity.

These tests provided the basis for five language domain scores that we used in our data analysis: single word production, single word comprehension, spelling, sentence comprehension, and sentence production. To obtain domain-specific severity scores, the proportion correct score for each domain was converted to a *z*-score based on the group mean and standard deviation, and the five *z*-scores were averaged to yield a composite language score for each participant. We correlated these domain and composite scores with *z*-transformed WAB-AQ scores (see Table 3 for scores by participant), with results showing strong correlations between measures: naming: *r*(33) = .85, *p* < .0001; word comprehension: *r*(33) = .77, *p* < .0001; spelling: *r*(31) = .71, *p* < .0001; sentence comprehension: *r*(33) = .61, *p* < .0001; sentence production: *r*(33) = .79, *p* < .0001; and composite language score: *r*(33) = .93, *p* < .0001. Spelling scores were not available for two participants.

### 2.2. Data Acquisition

Images were collected on four different 3.0T systems: a Siemens TIM Trio with a 32-channel head coil (Northwestern University), a Siemens Prisma with a 64-channel head/neck coil (Northwestern University), a Skyra with 20-channel head/neck coil (Boston University), and a Philips Intera with a 32-channel head coil (Johns Hopkins). Prior to the study, imaging sequences were equated across sites, using the same parameters in all scanners. Resting CBF maps were collected using a pseudo-continuous arterial spin labeling (pCASL) sequence [41] with two-dimensional gradient echo-planar readout (EPI): field of view (FOV) = 220 mm, in-plane resolution = 3.4 × 3.4 mm^2^, 25 slices, thickness = 4 mm with 1 mm gap, and TE/TR = 11 ms/4500 ms. The labeling plane was situated 90 mm below the center of the imaging volume, and labeling pulses were applied for 1.5 s. The postlabeling delay was set to 1900 ms to balance between potential slow flow and adequate signal to noise ratio [42]. Sixty pairs of interleaved control and tag images were acquired for signal averaging. In addition to the ASL scan, high resolution T1-weighted anatomical images were acquired using an MPRAGE sequence [43]: FOV = 256 mm, TE/TR/TI = 2.91 ms/2300 ms/900 ms, 176 sagittal slices, and resolution of 1 mm^3^.

### 2.3. Data Processing

Perfusion-weighted images from the pCASL scan were processed using a pipeline incorporating commands from Statistical Parametric Mapping (SPM8, Wellcome Trust Center for Neuroimaging, London, UK), and code developed in-house with Matlab R2013a (Mathworks, Natwick, MA) and implemented on the Northwestern University Neuroimaging Data Archive (NUNDA) [44]. Briefly, the raw EPI images were first aligned to the first image of the time-series to extract 6 motion-related measures for the time-series. The motion parameters and signal from voxels containing 99% CSF were regressed out of the time-series to remove motion-related and physiological fluctuations in the signal [45]. Perfusion-weighted time-series were generated using pairwise subtraction, and outliers were removed based on the following criteria [46]: (a) translation greater than 0.8 mm; (b) rotation greater than 0.8°; (c) global signal or noise greater than 2 times the standard deviation. An average of 7 pairs of images was discarded from each ASL scan based on these criteria. The final perfusion-weighted time-series were then converted into quantitative flow (*f*) maps in units of mL/100 g/min using the following equation:

(1) $$\begin{array}{c} f=\frac{\lambda \cdot \Delta M}{2\alpha M_{0}\cdot T_{1b}\cdot \left(e^{-PLD/T_{1b}}-e^{-\left(\tau +PLD\right)/T_{1b}}\right)}, \end{array}$$

where *λ* is the blood/tissue partition coefficient = 0.9 mL/g [47], Δ*M* is the perfusion-weighted signal, *α* is the inversion efficiency = 0.85 [41], *M*_0_ is the equilibrium signal of tissue, PLD is the post-labeling delay, *τ* is the labeling duration, and *T*_1*b*_ is the *T*_1_ of blood = 1664 ms [48].

Due to the low resolution of CBF maps, partial volume effects are prominent and need to be corrected before any further analysis. This was implemented as another NUNDA pipeline based on the following equation derived from positron emission tomography CBF studies [49]:

(2) $$\begin{array}{c} f_{GM}=\frac{f_{uncorr}-P_{WM}\cdot f_{WM}}{P_{GM}}, \end{array}$$

where *f*_uncorr_ is the uncorrected flow value, *P*_GM_ and *P*_WM_ denote gray and white matter probability in the voxel, extracted from tissue segmentation of the high resolution anatomical image, and *f*_GM_ and *f*_WM_ are the corresponding tissue-specific flow values. *f*_WM_ was extracted from voxels containing 99% white matter. To minimize artifactually high CBF due to division by small numbers, the above calculation was limited to voxels containing at least 30% gray matter. The partial volume corrected CBF maps were then spatially normalized to MNI space using the transformation matrix calculated from the high resolution anatomical image.

### 2.4. Lesion Volume

**Figure 1:**
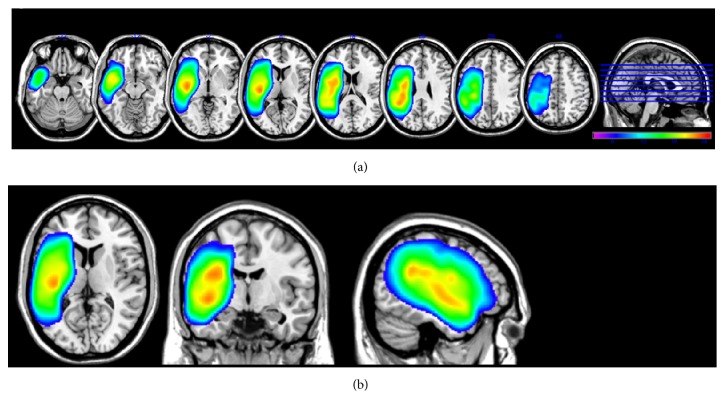
Lesion overlap map for 35 participants with aphasia, by axial slices (a) and with a three-dimensional view (b), using the neurological convention (left hemisphere is on the left). The color bar indicates the degree of overlap from minimal overlap (violet; *N* = 2 participants overlapping) to maximum overlap (red; *N* = 25 participants overlapping). The overlap map was spatially smoothed (3 mm).

Lesion volume was derived from lesion maps, developed by manual drawings measured using MRIcron software (http://www.sph.sc.edu/comd/rorden/mricron). To delineate the borders of necrotic tissue in each patient, we first determined intensity measures for white and gray matter (WM and GM, resp.) in the contralateral (right) hemisphere for each axial slice. The minimum right hemisphere WM intensity was determined. Left hemisphere lesioned tissue, on each slice, was drawn using the pen tool of MRIcron. Then the minimum WM intensity was applied to the outlined area using the intensity filter function. Additional manual correction was applied using lesion outlines in multiple corresponding coronal and sagittal views. Total lesion volume was calculated by summing the number of lesioned voxels in the left hemisphere for each participant. In our analyses the size of each voxel was 1 mm^3^ and therefore lesion volume is reported in mm^3^. Composite axial T1 MR images showing lesion location and overlap for the 35 participants in the study are shown in Figure 1.

### 2.5. Regions of Interest (ROIs)

ROIs were defined based on the Harvard Oxford atlas thresholded at minimum of 25% gray matter, as well as from the Automated Anatomical Labeling (AAL) atlas. The list of ROIs (*n* = 76, 38 per hemisphere) is given in Table 4. Second, two perilesional ROIs and their right hemisphere homologues were created by dilating the lesion to 6 mm (0–6 mm) and 12 mm (6–12 mm) beyond its boundaries and subtracting the original lesion volume.

Because CBF is a physiological parameter that fluctuates with many factors such as vasoactive agents in food, beverages, and drugs and varies widely between subjects, all CBF values were normalized to the mean CBF of each individual's right occipital lobe ROI, assuming that CBF in this region is not compromised by a left hemisphere stroke resulting in aphasia. Importantly, raw perfusion values in this region did not differ significantly between patients (M = 68.8, SD = 25.5) and controls (M = 77.6, SD = 18.0), based on a one-way ANCOVA adjusting for age (*F*(1,48) = 2.55, *p* = .12).

Mean CBF within each ROI was only computed from voxels with 30% or more gray matter, as these are the only voxels that survived the partial volume correction step detailed above. In addition to correcting for partial volume, the ROIs also accounted for the lesion mask (voxels where the lesion value is set to 1 were excluded) and the field of view of the perfusion scan (an FOV mask was created to exclude all voxels not covered by the perfusion scan). Thus lesions were excluded from the analysis, as otherwise they might substantially lower the CBF values in the left hemisphere. Indeed, mean CBF across participants in lesioned voxels was substantially lower (M = 17.95, SE = 2.1) than in nonlesioned voxels in the left hemisphere (M = 59.78, SE = 3.1).

### 2.6. Data Analyses

To test whether perfusion laterality differs broadly between patients and healthy controls, we conducted a 2 (hemisphere: left versus right as a within-subjects factor) × 2 (group: patient versus healthy control as a between-subjects factor) Repeated Measures Analysis of Covariance (RMANCOVA) with age as a covariate. Note that this analysis did not include all voxels from each hemisphere; rather, we included only the data from the 38 regions of interest (ROIs) that were the focus of our investigation. Follow-up tests were one-way ANCOVAs adjusting for age. The level of statistical significance in all inferential analyses (here and those described below) was *p* ≤ .05.

We also tested perfusion values from each of the ROIs (ROI: 76 levels, as a within-subjects factor) with group (patients versus controls) as a between-subjects factor and age as a covariate. Given the large number of ROIs and brain regions included in this analysis, we conducted analogues of protected *t*-tests to protect against inflated experimentwise Type I error [50]. Simulations have shown that this approach provides adequate Type I error rate protection, while affording better power than other approaches when conducting multiple tests [51]. In the present analysis, this approach consisted of using repeated measures analyses and only testing for group differences within individual regions if there was a significant ROI × group interaction. For the follow-up tests, we used one-way ANCOVA, adjusted for age, comparing patients against controls in each ROI.

#### 2.6.1. Perilesional ROI Analyses

Analyses of perilesional perfusion were also conducted for the patients only, with three RMANOVAs. First, a 2 (perilesional space: 0 to 6 mm versus 6 to 12 mm) × 2 (region: left perilesional versus right homologue) test was performed on data from 35 patients comparing perfusion in the perilesional area of the left hemisphere to a homologous contralateral area in the right hemisphere. A second analysis compared perfusion in perilesional space to that in the remainder of the left hemisphere (i.e., the remainder of the entire hemisphere, not restricted to the 38 ROIs in our other analyses) using a 2 (perilesional space: 0 to 6 mm versus 6 to 12 mm) × 2 (left hemisphere region: perilesional versus the remainder of the left hemisphere) RMANOVA. For the 0–6 mm perilesional space, the remainder of the left hemisphere excluded the lesion and the 0–6 mm space; for the 6–12 mm perilesional space the remainder excluded the lesion and perilesional tissue from 0 to 12 mm. The third analysis examined all three regions across both hemispheres using a 3 (perilesional space: 0–6 mm, 6–12 mm, and 12+ mm) × 2 (left versus right hemisphere) RMANOVA.

#### 2.6.2. Associations between Perfusion and Language and Demographic Variables

First, we computed a difference score for each of the 38 bilateral ROIs as well as perilesional ROIs (i.e., 0–6 mm and 6–12 mm), subtracting left hemisphere perfusion values from right hemisphere perfusion values, such that positive scores indicated lower perfusion in left hemisphere tissue compared to the right. These difference scores then were correlated with composite language scores with partial correlations adjusting for lesion volume. Given that we computed these partial correlations for 38 ROIs, we applied a Holm correction [52] for multiple comparisons. If the partial correlation was significant with the correction applied, we followed up with additional partial correlations between the perfusion difference score and each of the five language domain scores (word production, word comprehension, spelling, sentence comprehension, and sentence production). Finally, we computed partial correlations, adjusting for lesion volume, between mean perfusion values (normalized to the right occipital lobe) for each hemisphere separately (excluding the lesion and the 0–6 mm perilesional ROI) with language composite and domain scores and demographic variables (WAB-AQ, age, sex, education, and lesion age), and computed the simple correlation between perfusion and lesion volume itself.

## 3. Results

**Figure 2:**
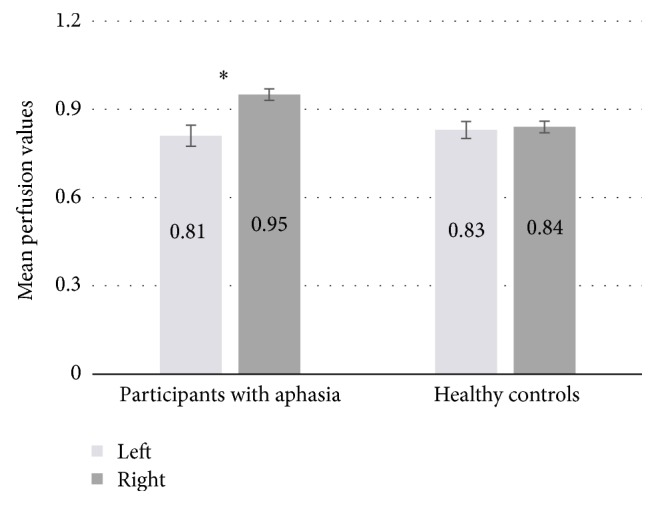
Mean right-occipital-normalized perfusion values for participants with aphasia and healthy controls, averaged across the 38 ROIs for the left and right hemispheres. Error bars are standard error. *∗* indicates a significant left versus right difference (*p* < .05).

The RMANCOVA examining hemisphere by group effects (including age as a covariate) showed a significant group × hemisphere interaction (*F*(1,48) = 11.27, *p* < .01) (Figure 2). Follow-up RMANOVAs demonstrated no significant difference between the perfusion values (over the 38 ROIs) for the left (M = .83, SD = .08) and right hemispheres (M = .84, SD = .08) in healthy control participants (*F*(1,14) = 1.62, *p* = .22); however, for the aphasic participants, perfusion values over the 38 ROIs in the right hemisphere (M = .95, SD = .17) were significantly higher than in the left hemisphere (M = .81, SD = .21) (*F*(1,33) = 4.02, *p* = .05). In addition, one-way ANCOVAs revealed a difference approaching conventional levels of significance with higher perfusion values for the patients compared to the healthy controls in the right (*F*(1,48) = 2.84, *p* < .10), but not in the left hemisphere (*F*(1,48) = .45, *p* = .51).

**Figure 3:**
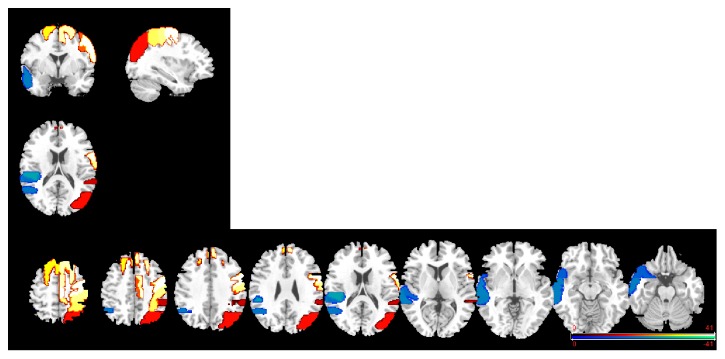
ROIs with greater perfusion (hyperperfusion; red-yellow color scale) and lesser perfusion (hypoperfusion; blue-green color scale) in patients relative to control participants, in three-dimensional and axial slice views (left hemisphere is on the left). Only regions that differ significantly across groups (patients versus controls; *p* < .05) are indicated.

The RMANCOVA examining perfusion differences between participant groups by ROI, with age as a covariate, revealed a significant interaction of group × ROI (*F*(75,3525) = 3.87, *p* < .001). As mentioned above, we used an analogue of protected *t*-tests to protect against inflated experimentwise Type I error. That is, for this set of analyses, if the group × ROI interaction had not been significant we would not have tested for group differences in each of the individual ROIs. Given that the interaction was significant, we proceeded by testing for group differences within individual ROIs. As shown in Table 4(b) and Figure 3, the groups differed significantly on 16 of the total 76 ROIs across hemispheres. Age-adjusted perfusion value differences between the patients and control participants in the left hemisphere were significant in nine ROIs, with lower values for patients in eight of these regions: the anterior and posterior superior and middle temporal gyri, the temporal pole, the angular gyrus, the planum temporale, and the parietal opercular cortex. The superior frontal gyrus showed the opposite pattern with higher values for patients. Age-adjusted perfusion value differences in the right hemisphere were significant in seven ROIs, all in the direction of higher perfusion for the patients: superior frontal gyrus, precentral gyrus, postcentral gyrus, superior parietal lobule, posterior supramarginal gyrus, superior lateral occipital cortex, and supplementary motor area (SMA).

### 3.1. Perfusion in Perilesional ROIs

The 2 (dilation of perilesional space: 0–6 mm versus 6–12 mm) × 2 (region: left perilesional versus right homologue) RMANOVA revealed a significant dilation × region interaction (*F*(1,34) = 52.60, *p* < .001) in the aphasic patient group. For the 0–6 mm dilation there was significantly greater perfusion in the homologous right hemisphere space (M = .99, SD = .23) than the left perilesional hemisphere region (M = .77, SD = .21; *t*(34) = 7.64, *p* < .001). Perfusion was also significantly greater for the 6–12 mm dilation in the homologous right hemisphere space (M = .97, SD = .21) than the left perilesional hemisphere region (M = .92, SD = .23; *t*(34) = 2.32, *p* = .027). The interaction reflects a larger left versus right difference for 0–6 mm than 6–12 mm.

The 2 (dilation of perilesional space: 0–6 mm versus 6–12 mm) × 2 (region: left perilesional versus the rest of the left hemisphere) RMANOVA revealed a significant dilation × region interaction (*F*(1,34) = 53.29, *p* < .001). Comparisons between the perilesional region and the rest of the left hemisphere (M = .94, SD = .22) was significant for the 0–6 mm ROI (M = .77, SD = .21) (*t*(34) = 5.62, *p* < .001), but not for the 6–12 mm perilesional region (M = .92, SD = .22) (*t*(34) = .34, ns). Perfusion also was significantly lower in the 0–6 mm ROI than in the 6–12 mm ROI (*t*(34) = 7.57, *p* < .0001).

**Figure 4:**
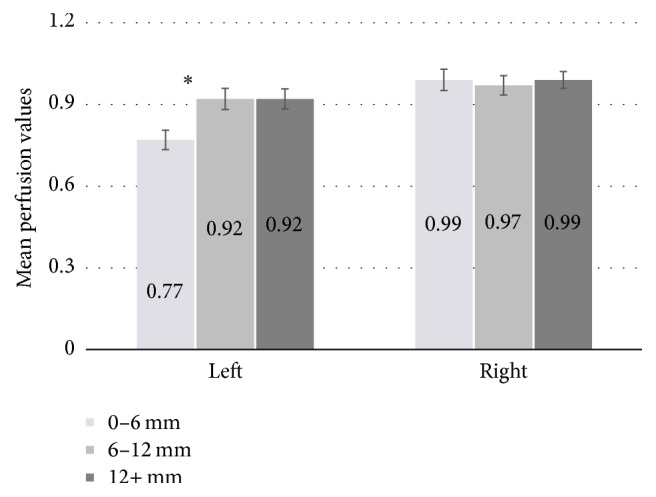
Mean right-occipital-normalized perfusion values for participants with aphasia for the left perilesional tissue and the corresponding right homologous regions in the 0–6 mm, 6–12 mm, and remaining (12+ mm) ROIs. Error bars are standard error. *∗* indicates a significant difference (*p* < .05). Significance is not indicated for left versus right differences (all ROIs are significant between hemispheres).

Finally, we conducted an overall 3 (dilation: 0–6 mm, 6–12 mm, rest of hemisphere) × 2 (hemisphere: left versus right) RMANOVA (Figure 4). The interaction between dilation and hemisphere was significant (*F*(2,68) = 34.8, *p* < .0001). Follow-up *t*-tests are consistent with the previous analyses, with perfusion in the left hemisphere lower than in the right hemisphere at each dilation (all *p*s < .05). In addition, within the left hemisphere, perfusion in the 0–6 mm region was lower than in the 6–12 mm region (*p* < .05), and perfusion was no different for the 6–12 mm region than the remaining left hemisphere tissue. No within-hemisphere contrasts reached significance in the right hemisphere.

### 3.2. Relationship between Perfusion, Language Performance, and Patient Variables

Correlational analyses for each ROI difference score (i.e., right minus left hemisphere perfusion values) and composite language scores, adjusting for lesion volume, showed significant negative correlations (i.e., greater difference scores and poorer language performance) in 6 ROIs: anterior inferior temporal gyrus (*r* = −.354, *p* = .04), postcentral gyrus (*r* = −.360, *p* = .037), supplementary motor area (SMA; *r* = −.419, *p* = .014), paracingulate gyrus (*r* = −.353, *p* = .04), anterior cingulate gyrus (*r* = −.344, *p* = .046), and posterior cingulate gyrus (*r* = −.360, *p* = .037). However, no correlation remained significant when the correction for multiple comparisons was applied. Accordingly, we did not follow up on these analyses with correlations between the perfusion difference scores and the five language domain scores.

With respect to the perilesional regions of interest, correlations between the composite language score and perilesional difference scores (i.e., right homologous perilesional ROI minus left perilesional ROI values), with lesion volume included as a covariate, revealed a significant negative correlation for 0–6 mm (*r* = −.469, *p* = .007), but not for 6–12 mm (*r* = −.288, *p* = .11). Thus, we calculated partial correlations with the five language domain scores separately only for the 0–6 mm perilesional ROI, adjusting for lesion volume. Results revealed significant negative associations between perfusion difference scores for the 0–6 mm ROI and single word production (*r* = −.354, *p* = .032), sentence comprehension (*r* = −.427, *p* = .015), and sentence production (*r* = −.451, *p* = .01). No other partial correlations reached significance (all *r*s ≤ |.30|, all *p*s ≥ .097). These effects are summarized in Table 5.

Finally, partial correlations (adjusting for lesion volume) between average perfusion values in nonperilesional tissue across each hemisphere (in the left, excluding the infarcted region and the 0–6 mm perilesional region; in the right, also excluding regions homologous to the lesion and the 0–6 mm perilesional ROI) and composite language scores for the patient group were not significant for the left hemisphere (*r* = .14, *p* = .45) or the right hemisphere homologous region (*r* = .004, *p* = .98). Likewise, correlations between average perfusion and demographic variables including age, sex, education, and lesion age (in months) revealed no significant correlations or partial correlations (correcting for lesion volume) for either hemisphere (all *r*s ≤ |.26|, all *p*s ≥ .12). However, a significant negative correlation between perfusion and lesion volume was found for the left (*r* = −.37, *p* = .027) but not the right hemisphere (*r* = −.27, *p* = .112).

## 4. Discussion

This paper examined perfusion values, normalized to the right occipital lobe, in people with chronic stroke-induced aphasia compared to cognitively healthy, right-handed, non-brain-damaged control participants. We focused our investigation on 38 regions of interest in each hemisphere. Results showed that whereas healthy controls evince no significant between-hemisphere differences in normalized perfusion values, averaged across our ROIs, the aphasic participants' values differ significantly between the left and right hemisphere. However, rather than showing left (ipsilesional) hemisphere hypoperfusion, as predicted, the patients showed normalized perfusion values similar to healthy controls in the left hemisphere, with no significant difference found between the two participant groups. Conversely, the aphasic group showed hyperperfusion in the right (contralesional) hemisphere, with overall perfusion values significantly greater compared to controls. Furthermore, for the aphasic group, right hemisphere perfusion was significantly higher than left hemisphere perfusion. These findings are broadly consistent with those reported by Richardson et al. [11], who found lower perfusion values in the left compared to the right hemisphere in participants with aphasia. However, patient perfusion values were not compared to a healthy control group, precluding the finding that between-hemisphere differences may have resulted from greater than normal right hemisphere perfusion in their patient group rather than lesser than normal left hemisphere perfusion.

Notably, not all regions in the left hemisphere were normally perfused in the patient group, and not all regions in the right hemisphere were hyperperfused. Within the left hemisphere, 8 regions showed a pattern of significant hypoperfusion, and one region showed increased perfusion. The remaining 29 regions did not differ between patients and controls. The lack of an overall effect of left hemisphere hypoperfusion likely reflects this variability, such that focal hypoperfusion was averaged out across the full set of 38 ROIs. In the right hemisphere, perfusion was significantly higher in the patient group compared to healthy controls in 7 regions, but no right hemisphere regions were hypoperfused. The remaining 31 regions did not differ significantly between patients and controls.

Note that this pattern of variable hypo- and hyperperfusion does not appear to be a consequence of our decision to normalize the raw perfusion values to the right occipital lobe. First, the raw perfusion values for the participants with aphasia and the healthy controls did not differ significantly in this region, suggesting that normalization did not introduce a systematic bias across groups.

One interpretation of the unexpected finding of hyperperfused regions in the right hemisphere is that autoregulation of blood flow is adaptive to vascular lesion, with upregulation in undamaged regions. Blood typically directed automatically, for example, to the left hemisphere middle cerebral artery (MCA), is shifted elsewhere, potentially to the left anterior cerebral artery (ACA) or the right MCA. If this were the case, however, we might expect all tissue supplied by these vessels to show equally greater perfusion, and perfusion in these regions would putatively be higher than that in regions supplied by other sources (e.g., the posterior cerebral artery (PCA)).

Although we did not examine every region supplied by these blood vessels, there may nonetheless be a pattern along these lines. In the left hemisphere, all of the regions found to be significantly hypoperfused are supplied by the MCA: the anterior and posterior superior and middle temporal gyri, temporal pole, angular gyrus, planum temporale, and parietal opercular cortex, whereas one left hemisphere region found to be hyperperfused is supplied by the ACA: the superior frontal gyrus. Regions supplied by the PCA were not abnormally perfused in either hemisphere. Furthermore, there were no hypoperfused regions supplied by the ACA and no hyperperfused regions supplied by the MCA or PCA. Thus, the overall pattern in the left hemisphere seems to be that, among the regions we examined, the regions supplied by the MCA are hypoperfused (or normal) and those supplied by the ACA are hyperperfused or normal, but regions supplied by the PCA show normal perfusion levels.

In the right hemisphere, regions either were normally perfused or showed perfusion levels significantly greater than that of the healthy controls. Of the (significantly) hyperfused regions, one is supplied by the MCA (the posterior supramarginal gyrus), three are supplied by the ACA (superior frontal gyrus, superior parietal lobule, and supplementary motor area), two are supplied by both the ACA and MCA (precentral and postcentral gyri, which are supplied by the ACA medially and the MCA laterally; our perfusion measures did not distinguish medial versus lateral aspects of these regions), and one is supplied by the PCA (superior lateral occipital cortex). The pattern in the right hemisphere thus appears complementary to the pattern in the left; that is, regions supplied by the MCA are hyperperfused (or normal). Similarly, as in the left hemisphere, hyperperfused regions are supplied by the ACA and regions supplied by the PCA are largely normal.

This appears to be consistent with a compensatory change leading to increased perfusion in regions supplied by the right MCA and bilateral ACA in response to reduced perfusion in regions supplied by the left MCA and may reflect right hemisphere vascular reserve engaged to absorb and distribute additional blood flow. However, this is not clear-cut in that hypoperfused regions in the left were not hyperperfused in the right hemisphere (except for the anterior superior temporal gyrus, which was significantly hypoperfused in the left hemisphere with hyperperfusion that approached significance in the right).

The functional significance of hyperperfusion in regions within the right hemisphere is also not completely clear. One interpretation is that this reflects maladaptive language processing, although correlations between perfusion difference scores (right-left hemisphere) and language performance (i.e., greater right hemisphere perfusion and poorer language ability) were not significant when corrected for multiple comparisons. Thus, it is unlikely that right hemisphere hyperperfusion alone reflects inefficient language function. Another more likely interpretation is that, because increased perfusion reflects increases in neuronal energy usage, perfusion value increases in our patients may be associated with generally increased cognitive effort. By virtue of a left hemisphere lesion, right hemisphere regions become more actively engaged. This interpretation is also supported by our observed bilateral hyperperfusion in the SMA (though the increased perfusion only approached significance in the left hemisphere). The SMA is one of several domain-general cognitive regions associated with the multiple-demand system in healthy people, which is engaged for language and other cognitive tasks when domain-specific resources are disrupted or unavailable [53, 54]. Notably, the pattern of hyperperfused regions is also in line with the Scaffolding Theory of Cognitive Aging (STAC) [55], which suggests that bilateral frontal regions (i.e., superior frontal and SMA) are engaged as a function of aging to compensate for neurocognitive decline and may also be available when brain damage compromises cognitive ability. Our results encourage further investigation in this direction.

When the brain is divided into regions based on rings of perilesional tissue, the results are less unexpected. Our findings showed that, on average, for perilesional areas, patients had significantly lower perfusion values in the left hemisphere than in homologous regions in the right hemisphere. However, within the left hemisphere, perfusion values became more normal in our participants with increasing distance from the lesion. Thus, even in chronic stages of aphasia a perilesional ring close to the lesion remains substantially hypoperfused. Importantly, relative perfusion values in the 0–6 mm (but not 6–12 mm) perilesional region correlated with language severity, even when accounting for lesion volume. The lesion-adjacent region may therefore not only have a greater reduction in cerebral blood flow, but the extent of reduced blood flow in this region is also predictive of language impairment. For our participant group, perilesional perfusion (0–6 mm only) was significantly correlated with naming, sentence comprehension, and sentence production. We note, however, that this latter finding may reflect the language impairment patterns of our aphasic participants in that the majority of our participants were selected for naming impairments (*n* = 21 from Boston University), with 11 selected for impaired sentence production and comprehension (from Northwestern) and 5 selected for dysgraphia (from Johns Hopkins).

We note, however, that while our results speak to the importance of lesion-adjacent perilesional tissue for impaired language, we did not attempt to determine a precise boundary within which tissue may be underperforming, and beyond which tissue may be functioning normally. There is unfortunately no standard operational definition of what constitutes “perilesional” tissue [20]. Some previous studies have identified hypoperfused tissue in a 3–8 mm ring around the lesion [11], whereas others have reported reduced perfusion as far away as 15 mm from the lesion [20]. The problem here is twofold: an objectively determined anatomical method for determining hypoperfused tissue has not been identified, and any such method needs to account not only for fine-grained differences across brain regions (e.g., at the voxel level), but also for the possibility that perilesional rings may not adequately capture the functional impact of vascular lesions. Depending on the volume and location of the lesion, tissue surrounding it may include both normally perfused and hypoperfused tissue, such that averaging perfusion within the entire ring may lead to spurious results. It is possible, for example, that ribbons of hypoperfused tissue, extending distally from the lesion and including both perilesional and other cortical tissue, may better capture the cognitive effects of brain damage. In addition, lesion-adjacent rings may often include neural tissue that was involved in language processing prior to stroke as well as tissue that was not, thus, precluding determination of a clear relation between perfusion and language impairment. Further research is needed to identify the functional significance of reduced perfusion at various distances from the lesion, in particular regions within lesion-adjacent tissue, and in pathways following the vasculature.

Finally, we note that the only nonlanguage measure that correlated with perfusion in the remaining (undamaged and nonperilesional) portion of the left hemisphere was lesion volume. However, no correlation between lesion volume and perfusion in the right hemisphere region was found. Likewise, no correlations were found between perfusion (in either hemisphere) and lesion age (months after onset of stroke), chronological age, education, or sex. These results are consistent with prior findings in the literature and suggest that perfusion levels reach a stable steady state in individuals with chronic stage aphasia and are also not associated with general demographic variables.

While our results showed patterns of both hypoperfusion and hyperperfusion in chronic aphasia and link some of these perfusion changes to impaired language, questions remain. We did not address what this means for recovery of language in chronic stages of aphasia. With respect to hypoperfusion, the point at which reduced cerebral blood flow results in functional deficiencies or is indicative of a nonreversible state is unknown. Although animal models suggest that perfusion levels below 30% of normal constitute hibernating, nonfunctional tissue, we found correlations between perfusion and language impairment in the 0–6 mm perilesional ROI, where the mean normalized perfusion value was just below 80%. Correspondingly, we also do not know if hyperperfusion reflects language inefficiency, or what levels of perfusion impair (or improve) cognitive function. The regions with the greatest levels of right hemisphere perfusion were not consistently or significantly associated with language disability, and none of the right-occipital-normalized values within the ROIs we examined were more than 40% above those of the normal control participants. The time course by which certain regions of the brain become hyperperfused also is not known. It is possible that heightened perfusion levels in contralesional regions are an immediate consequence of stroke, though it could also be the case that such changes develop slowly over time, possibly reflecting attempts to compensate for left hemisphere brain damage.

## 5. Conclusions

In summary, we report two key findings regarding perfusion in chronic aphasia. First, we found a varied pattern of hypoperfused and hyperperfused regions across both the left and right hemispheres of the brain. These patterns suggest that autoregulatory shifts in blood flow in response to lesions within the distribution of the left middle cerebral artery may be associated with the abnormal perfusion patterns we observed; however this possibility requires further investigation. Notably, our findings do not strongly support the idea that perfusion changes (in particular right hemisphere hyperperfusion) reflect maladaptive language processing. Rather, we suggest that regions of increased perfusion reflect changes in domain-general cognitive effort. Secondly, we found that perilesional tissue within 6 mm of the lesion is particularly hypoperfused compared to regions more distal to the lesion. Importantly, the degree of hypoperfusion in this perilesional region correlates with performance on standard measures of language ability, when adjusting for lesion volume, with reduced perfusion corresponding to more impaired language. Critically, however, we suggest that perilesional rings may only crudely capture the effects of vascular lesions on perfusion due to heterogeneity of lesion location and volume as well as variability in the properties of lesion-adjacent tissue. Finally, the present results underscore the need to consider chronically altered cerebral blood flow as a contributing factor to the persistent language deficits in chronic aphasia, which might also serve as an additional avenue for targeted recovery of language function.

## Figures and Tables

**Table 1:** Studies of perfusion in chronic aphasia.

Study	Sample size (*n*)	Time since stroke	Diagnosis	Treatment protocol	MRI method	Task	Key findings
Love et al., 2002	1	16 years	Anomia, difficulty in reading	—	PASL	Resting state	(i) Hypoperfusion in L angular gyrus, L supramarginal gyrus; neither region infarcted

Peck et al., 2004	3	8–48 months	Nonfluent aphasia	2 with intention treatment; 1 with attention treatment	BOLD TTP	Category member generation	(i) From pre- to posttreatment, average difference across patients in TTP between R auditory cortex and R motor cortex decreased, corresponding to shortened posttreatment response times, and approached the average value for controls

Fridriksson et al., 2006	1	18 months	Aphasia (incl. moderate anomia)	—	PWI/BOLD	Overt picture naming	(i) Delayed TTP in resting state PWI in LH versus RH (ii) Abnormal HRF in activated areas during naming

Bonakdarpour et al., 2007	5	>2 years	Agrammatic aphasia	—	BOLD TTP	Lexical decision	(i) Increased TTP in L perisylvian cortex (3 of 5 individuals) relative to healthy controls (ii) No differences in R perisylvian or L or R occipital cortex

Brumm et al., 2010	3	2–11 years	Expressive aphasia	—	PASL	Resting state	(i) Hypoperfusion in L penumbra (2 voxels); noninfarcted regions of L hemisphere

Thompson et al., 2010	6	6–146 months	Agrammatic aphasia	Treatment of Underlying Forms (TUF)	PASL	Resting state	(i) Regions with upregulated BOLD response (auditory sentence-picture verification task) following treatment showed faster TTP (ii) After treatment, 4 patients decreased TTP in L angular gyrus; 3 decreased TTP in L superior parietal cortex; 4 decreased TTP in R superior parietal cortex

Richardson et al., 2011	17	4–246 months	Aphasia (not specified)	—	PASL	Resting state	(i) Hypoperfusion in L penumbra (8 mm); noninfarcted regions of L hemisphere (ii) Larger lesion correlated with reduced perfusion

Fridriksson et al., 2012	30	6–350 months	13 Broca's; 10 anomic; 3 conduction; 2 Wernicke's; 1 Trans-cortical motor; 1 global	Anomia treatment	PASL	Resting state	(i) Pretreatment perfusion levels in residual language network regions, that is, not infarcted and not perilesional (15 mm), predicted posttreatment improvement in picture naming (ii) Pretreatment perfusion levels in infarcted and perilesional tissue did not predict posttreatment improvement

Bonakdarpour et al., 2015	5	6–96 months	2 Broca's aphasia; 3 anomia	—	BOLD TTP	Overt picture naming	(i) Increased TTP in L hemisphere naming regions relative to healthy controls (ii) No difference in percent signal change

PASL: pulsed arterial spin labeling; PWI: perfusion weighted imaging; TTP: time to peak (of the hemodynamic response function (HRF)); SMA: supplementary motor area.

**Table 2:** Participant information (mean and standard deviation).

Group	Age (years)	Sex	Education (years)	Lesion age (months)	WAB-AQ^1^
Aphasia (*n* = 35)	57.7 (10.5)	21 M/14 F	15.8 (2.1)	59.3 (53.0)	66.2 (22.7)
Controls (*n* = 16)	32 (8.5)	8 M/8 F	17.7 (1.7)	—	—

^1^WAB-AQ: Western Aphasia Battery-Aphasia Quotient.

**Table 3:** WAB Aphasia Quotients (AQ), language domain scores, and composite language scores (as *z*-scores) for each participant with aphasia.

Participant	WAB-AQ^1^	Composite language	Naming	Spelling	Word comprehension	Sentence comprehension	Sentence production
BU01	.92	.74	1.18	.33	.54	.41	1.23
BU02	−1.81	−1.39	−1.64	−1.20	−2.45	−.56	−1.12
BU03	−.63	−.55	−1.19	−.63	.54	−.56	−.93
BU04	.35	.25	.84	1.38	.29	−.75	−.54
BU06	.02	−.35	.39	−.63	.54	−.94	−1.12
BU07	−.80	−1.07	−.74	−1.12	−.46	−1.91	−1.12
BU09	1.28	1.30	1.07	1.78	.54	1.39	1.72
BU10	.63	.61	.73	.89	.29	1.00	.15
BU11	1.14	1.11	.84	−1.20	.29	1.77	1.53
BU12	−1.15	−.99	−.85	−1.20	−1.20	−.56	−1.12
BU13	1.17	1.42	1.18	1.78	.54	1.77	1.82
BU14	−.08	.16	−.40	.97	.54	−.56	.25
BU15	.92	.11	.84	−1.12	.54	.03	.25
BU17	.36	.02	1.07	−.63	.54	−.56	−.34
BU18	.52	.49	.39	.25	.29	1.39	.15
BU20	−2.34	−1.26	−1.64	−1.04	−1.20	−1.31	−1.12
BU21	−2.40	−1.78	−1.64	−1.20	−4.20	−.73	−1.12
BU22	−.04	.14	−.73	.57	−.46	1.19	.14
BUc01	.85	1.08	1.07	1.46	.54	1.58	.74
BUc04	1.11	1.23	1.18	1.05	.54	1.77	1.62
BUc05	−1.49	−1.00	−1.64	−1.12	−.95	−.17	−1.12
JH06	1.00	.46	−1.08	1.46	.29	1.00	.64
JHc04	−1.02	−.46	−1.07	−.47	.54	−.17	−1.12
JHc05	−.38	−.45	−.17	−1.04	.04	.03	−1.12
JHc06	1.03	.48	.05	.17	.54	−.17	1.82
JHc07	.41	.22	.28	1.05	.29	.03	−.54
NU03	.42	.56	.84	.57	.54	.03	.84
NU04	−.56	−.09	−.74	−.39	.29	−.36	.74
NU05	.35	−.10	.39	−.23	.54	−.94	−.24
NU06	1.00	.46	1.29	−.47	.54	.41	.55
NU08	−.59	−.58	−.51	−1.12	.29	−.56	−1.03
NU13	−.29	−.65	−.63	−1.20	−.71	−1.53	.25
NUc01	.44	.41	1.18	−.07	.54	−.17	.55
NUc02	.22	.05	.84	.81	.54	−1.33	−.63
NUc03	−.56	−.48	−.97	−.87	.04	.03	−.63

^1^WAB-AQ: Western Aphasia Battery-Aphasia Quotient.

**(a):** 

Region of interest (ROI)	Left hemisphere			Right hemisphere		
	Controls	Patients	% diff	Controls	Patients	% diff
Inferior frontal gyrus, orbital part^1^	74.75 (12.84)	59.98 (23.92)	80%	78.07 (14.77)	71.53 (22.73)	92%
Frontal pole	75.04 (14.11)	68.46 (25.16)	91%	77.08 (16.00)	73.40 (25.03)	95%
Superior frontal gyrus	60.75 (16.51)	67.09 (28.58)	110%	62.66 (17.06)	74.22 (26.20)	118%
Middle frontal gyrus	68.85 (14.09)	62.28 (26.49)	90%	71.39 (16.35)	76.07 (29.97)	107%
IFG, pars triangularis	76.61 (15.53)	58.49 (29.30)	76%	82.37 (20.38)	71.97 (24.18)	87%
IFG, pars opercularis	72.03 (19.32)	50.57 (25.74)	70%	72.87 (19.82)	70.91 (23.61)	97%
Precentral gyrus	62.83 (13.81)	63.10 (22.87)	100%	63.90 (12.72)	77.83 (26.56)	122%
Temporal pole	69.53 (9.78)	51.17 (19.35)	74%	72.23 (10.69)	62.88 (18.99)	87%
Superior temporal Gyrus, anterior	60.67 (19.02)	41.56 (23.52)	69%	62.89 (22.44)	62.60 (23.19)	100%
Superior temporal gyrus, posterior	69.25 (16.40)	45.70 (19.07)	66%	71.41 (17.42)	68.82 (23.58)	96%
Middle temporal gyrus, anterior	65.34 (14.08)	46.73 (25.43)	72%	70.86 (16.02)	61.06 (21.72)	86%
Middle temporal gyrus, posterior	71.00 (14.54)	50.23 (26.53)	71%	73.47 (14.60)	62.30 (24.03)	85%
Inferior temporal gyrus, anterior	53.68 (16.01)	44.55 (26.86)	83%	53.61 (12.95)	45.52 (24.79)	85%
Inferior temporal gyrus, posterior	62.21 (16.40)	48.26 (19.22)	78%	54.44 (10.75)	50.62 (21.34)	93%
Inferior temporal gyrus, temporooccipital part	62.11 (12.94)	47.26 (22.41)	76%	67.39 (16.01)	53.72 (17.41)	80%
Postcentral gyrus	65.27 (14.88)	61.51 (21.92)	94%	65.52 (13.60)	76.04 (23.83)	116%
Superior parietal lobule	64.03 (14.48)	57.39 (22.92)	90%	60.59 (14.89)	69.38 (25.58)	115%
Supramarginal gyrus, anterior	65.10 (16.58)	47.49 (18.24)	73%	63.63 (13.51)	63.00 (21.94)	99%
Supramarginal gyrus, posterior	69.63 (17.40)	48.53 (21.63)	70%	68.57 (12.42)	66.65 (23.02)	97%
Angular gyrus	68.04 (16.70)	46.28 (25.04)	68%	65.94 (11.83)	66.88 (23.02)	101%
Lateral occipital cortex, superior	72.13 (18.07)	60.48 (24.44)	84%	73.05 (14.42)	73.41 (25.21)	100%
Lateral occipital cortex, inferior	77.21 (19.52)	59.67 (36.35)	77%	75.67 (18.08)	70.25 (27.28)	93%
Frontal medial cortex	68.35 (18.82)	57.89 (24.42)	85%	70.51 (18.57)	62.23 (24.21)	88%
Supplementary motor area (SMA)	59.08 (18.25)	62.57 (29.89)	106%	57.70 (16.41)	68.00 (24.91)	118%
Paracingulate gyrus	62.97 (13.94)	55.45 (18.16)	88%	66.20 (15.71)	61.56 (21.89)	93%
Anterior cingulate	61.74 (15.48)	55.41 (18.20)	90%	62.87 (15.38)	59.95 (19.52)	95%
Posterior cingulate	69.11 (16.05)	61.06 (23.29)	88%	70.68 (17.48)	67.99 (23.89)	96%
Precuneus	63.33 (17.26)	57.12 (22.06)	90%	63.91 (18.19)	63.07 (22.55)	99%
Parahippocampal gyrus, posterior	56.73 (24.49)	48.47 (17.98)	85%	56.53 (24.01)	55.70 (19.94)	99%
Temporal fusiform cortex, posterior	46.23 (10.08)	47.19 (18.14)	102%	45.85 (10.27)	46.93 (18.16)	102%
Temporal occipital fusiform cortex	49.67 (15.59)	45.64 (21.72)	92%	49.40 (12.85)	51.23 (21.74)	104%
Occipital fusiform gyrus	61.41 (16.06)	54.07 (29.00)	88%	61.51 (15.54)	58.79 (26.45)	96%
Frontal operculum cortex	60.12 (13.76)	37.58 (25.39)	63%	58.17 (12.15)	58.39 (21.55)	100%
Parietal operculum cortex	63.33 (14.96)	37.37 (16.72)	59%	61.32 (15.02)	59.44 (19.41)	97%
Planum temporale	75.43 (19.61)	50.39 (29.98)	67%	72.83 (19.65)	69.98 (24.90)	96%
Hippocampus	52.66 (10.83)	49.05 (17.47)	93%	54.57 (10.63)	50.49 (18.15)	93%
Cerebellum V	48.40 (19.89)	44.93 (17.66)	93%	50.52 (19.65)	41.83 (17.54)	83%
Cerebellum VI	54.17 (17.05)	47.90 (19.77)	88%	54.07 (15.33)	46.84 (18.76)	87%

**(b):** 

Region of interest (ROI)	Left hemisphere					Right hemisphere				
	Controls	Patients	*F*	*p*	% diff	Controls	Patients	*F*	*p*	% diff
Inferior frontal gyrus, orbital part^1^	.98 (.15)	.95 (.44)	.40	ns	97%	1.02 (.15)	1.10 (.33)	.85	ns	107%
Frontal pole	.98 (.13)	1.05 (.37)	.21	ns	107%	1.00 (.10)	1.12 (.32)	1.49	ns	112%
Superior frontal gyrus	**.79 (.16)**	**1.03 (.42)**	**4.12**	**.048**	**131%**	**.81 (.16)**	**1.12 (.34)**	**7.91**	**.01**	**138%**
Middle frontal gyrus	.90 (.13)	.95 (.36)	.98	ns	106%	.93 (.14)	1.14 (.34)	3.12	.08	123%
IFG, pars triangularis	1.01 (.20)	.93 (.44)	.00	ns	92%	1.06 (.15)	1.11 (.39)	.93	ns	104%
IFG, pars opercularis	.93 (.16)	.80 (.41)	.16	ns	86%	.94 (.14)	1.08 (.34)	1.89	ns	115%
Precentral gyrus	.82 (.11)	.98 (.37)	1.90	ns	120%	**.83 (.12)**	**1.18 (.33)**	**10.87**	**.002**	**141%**
Temporal pole	**.92 (.17)**	**.78 (.27)**	**5.32**	**.03**	**85%**	.96 (.20)	.97 (.28)	.61	ns	100%
Superior temporal gyrus, anterior	**.78 (.17)**	**.62 (.30)**	**6.74**	**.01**	**80%**	.80 (.19)	.95 (.30)	3.93	.05	118%
Superior temporal gyrus, posterior	**.91 (.17)**	**.71 (.29)**	**8.42**	**.01**	**79%**	.94 (.19)	1.04 (.27)	.84	ns	111%
Middle temporal gyrus, anterior	**.86 (.17)**	**.69 (.28)**	**6.97**	**.01**	**80%**	.93 (.16)	.93 (.28)	.06	ns	100%
Middle temporal gyrus, posterior	**.93 (.15)**	**.75 (.29)**	**7.13**	**.01**	**80%**	.96 (.14)	.93 (.23)	.06	ns	96%
Inferior temporal gyrus, anterior	.71 (.21)	.67 (.36)	.00	ns	94%	.72 (.21)	.67 (.28)	.07	ns	93%
Inferior temporal gyrus, posterior	.81 (.17)	.74 (.29)	.21	ns	91%	.72 (.16)	.77 (.27)	.05	ns	107%
Inferior temporal gyrus, temporooccipital part	.81 (.11)	.71 (.23)	.17	ns	87%	.88 (.13)	.80 (.16)	.00	ns	92%
Postcentral gyrus	.85 (.13)	.97 (.40)	.76	ns	114%	**.86 (.14)**	**1.16 (.34)**	**10.83**	**.002**	**136%**
Superior parietal lobule	.83 (.13)	.89 (.39)	.12	ns	107%	**.78 (.14)**	**1.05 (.35)**	**8.28**	**.01**	**134%**
Supramarginal gyrus, anterior	.84 (.13)	.76 (.34)	.72	ns	90%	.83 (.14)	.95 (.25)	2.61	ns	114%
Supramarginal gyrus, posterior	.90 (.13)	.76 (.33)	3.94	.05	84%	**.90 (.15)**	**1.01 (.26)**	**4.06**	**.049**	**112%**
Angular gyrus	**.88 (.14)**	**.72 (.37)**	**5.29**	**.03**	**81%**	.87 (.13)	1.01 (.28)	1.35	ns	117%
Lateral occipital cortex, superior	.93 (.09)	.93 (.34)	.08	ns	100%	**.95 (.10)**	**1.10 (.26)**	**4.25**	**.045**	**116%**
Lateral occipital cortex, inferior	.99 (.07)	.85 (.30)	3.64	.06	86%	.98 (.07)	1.02 (.14)	.17	ns	105%
Frontal medial cortex	.88 (.18)	.88 (.35)	.05	ns	100%	.91 (.17)	.95 (.31)	.14	ns	104%
Supplementary motor area (SMA)	.77 (.20)	.96 (.44)	2.84	.10	125%	**.75 (.17)**	**1.03 (.34)**	**6.96**	**.01**	**138%**
Paracingulate gyrus	.82 (.13)	.84 (.24)	.97	ns	103%	.86 (.14)	.92 (.23)	1.26	ns	107%
Anterior cingulate	.80 (.12)	.85 (.27)	.61	ns	106%	.82 (.13)	.91 (.25)	2.12	ns	111%
Posterior cingulate	.90 (.13)	.92 (.31)	.03	ns	103%	.91 (.13)	1.02 (.29)	.68	ns	112%
Precuneus	.82 (.12)	.86 (.24)	.38	ns	105%	.82 (.12)	.93 (.20)	1.04	ns	114%
Parahippocampal gyrus, posterior	.73 (.25)	.73 (.21)	.23	ns	100%	.73 (.27)	.84 (.25)	.02	ns	114%
Temporal fusiform cortex, posterior	.60 (.09)	.71 (.20)	1.84	ns	117%	.60 (.09)	.70 (.18)	.01	ns	117%
Temporal occipital fusiform cortex	.64 (.12)	.66 (.20)	.41	ns	104%	.64 (.11)	.74 (.14)	.06	ns	116%
Occipital fusiform gyrus	.79 (.12)	.79 (.28)	.05	ns	99%	.79 (.11)	.85 (.22)	.02	ns	107%
Frontal operculum cortex	.79 (.15)	.60 (.41)	3.91	.05	77%	.76 (.12)	.89 (.26)	3.01	.09	117%
Parietal operculum cortex	**.83 (.16)**	**.61 (.33)**	**6.48**	**.01**	**74%**	.80 (.18)	.91 (.26)	.47	ns	114%
Planum temporale	**.98 (.18)**	**.81 (.53)**	**5.54**	**.02**	**83%**	.95 (.19)	1.06 (.30)	.01	ns	112%
Hippocampus	.70 (.17)	.75 (.27)	.48	ns	106%	.72 (.17)	.76 (.21)	.26	ns	105%
Cerebellum V	.61 (.14)	.66 (.14)	.75	ns	108%	.64 (.15)	.62 (.15)	.36	ns	96%
Cerebellum VI	.69 (.10)	.71 (.18)	.17	ns	103%	.70 (.11)	.70 (.24)	.68	ns	101%

Bold cells: significant group differences (*p* < .05).

^1^Region from Automated Anatomical Labeling (AAL) atlas; all others from Harvard-Oxford Atlas.

% diff: percentage of normal (control participants) perfusion values for people with aphasia by ROI.

*Note*. Means and standard deviations of normalized perfusion values. *F* and *p* values derived from one-way ANCOVA, with age as a covariate.

**Table 5:** Partial correlations, controlling for lesion volume, between perilesional perfusion and language ability for 35 participants with aphasia.

Partial correlations controlling for lesion volume												
Difference Right-Left	Composite language		Naming		Spelling		Word comprehension		Sentence comprehension		Sentence production	
	*r*	*p*	*r*	*p*	*r*	*p*	*r*	*p*	*r*	*p*	*r*	*p*
0–6 mm	−.469^*∗*^	.007	−.354^*∗*^	.032	−.271	.13	−.299	.097	−.427^*∗*^	.015	−.451^*∗*^	.01
6–12 mm	−.288	.11	na		na		na		na		na	

*Note*. Difference scores were created by subtracting average perfusion in the perilesional area (left hemisphere) from the average perfusion in the analogous right hemisphere area. ^*∗*^*p* < .05.
